# Spatio-Temporal Roles of ASD-Associated Variants in Human Brain Development

**DOI:** 10.3390/genes11050535

**Published:** 2020-05-11

**Authors:** Yujin Kim, Joon-Yong An

**Affiliations:** 1 School of Biosystem and Biomedical Science, College of Health Science, Korea University, Seoul 02841, Korea; kyujin9805@korea.ac.kr; 2 Department of Integrated Biomedical and Life Science, Korea University, Seoul 02841, Korea

**Keywords:** autism spectrum disorders, autism spectrum disorder (ASD), neurodevelopment, systems biology, whole-exome sequencing, multi-omics analysis, gene pathway

## Abstract

Transcriptional regulation of the genome arguably provides the basis for the anatomical elaboration and dynamic operation of the human brain. It logically follows that genetic variations affecting gene transcription contribute to mental health disorders, including autism spectrum disorder (ASD). A number of recent studies have shown the role of *de novo* variants (DNVs) in disrupting early neurodevelopment. However, there is limited knowledge concerning the role of inherited variants during the early brain development of ASD. In this study, we investigate the role of rare inherited variations in neurodevelopment. We conducted co-expression network analyses using an anatomically comprehensive atlas of the developing human brain and examined whether rare coding and regulatory variants, identified from our genetic screening of Australian families with ASD, work in different spatio-temporal functions.

## 1. Introduction

The transcriptome generally refers to the set of Ribonucleic acid (RNA) molecules transcribed from nuclear Deoxyribonucleic acid (DNA). Transcription is a complex process governed by genetic regulatory elements that have evolved to spatially and temporally control the development of an organism. Transcription is further organized to respond to environmental cues and dynamically regulate the connection of the brain via constant cell remodeling of neurons. Consequently, an analysis of the temporal profile of gene regulation helps us understand the molecular mechanisms underlying cellular differentiation, development, or function of the brain [1,2,3,4,5]. When examining gene expression patterns comparing different biological groups (e.g., young vs. old brains; tissue A vs. tissue B, or disease-affected vs. unaffected), differences between RNA transcriptomes help to characterize the molecular basis of neurological conditions, phenotypes and disorders [6,7,8]. Over the past five years, several studies have used microarray and RNA-sequencing approaches to examine the transcriptomes of post-mortem human brain tissues. There is now a comprehensive transcriptome profile of the developing human brain from fetal through to adult stages [9,10]. Two resources from BrainSpan [1] have the most comprehensive gene expression data from brain tissue samples and include the largest data collection of brain structures and age groups [7,8].

Kang et al. surveyed the transcriptomes of 16 brain regions comprising 11 neocortex areas, cerebellar cortex, mediodorsal nucleus of the thalamus, striatum, amygdala, and hippocampus [7]. This data set is comprised of 1340 brain tissues, which were collected from 57 post-mortem brains from clinically “unremarkable” male and female donors. The post-mortem brains encompass fetal developmental stages (four post-conception week (PCW) stages), neonatal stages, childhood stages, and adults up to 82 years of age. Transcriptome data was generated using the Affymetrix Gene Chip Human Exon 1.0 ST Array platform, which contains 1.4 million probe sets and the survey’s expression of complete gene transcripts or individual exons. By using stringent quality control measures and criteria to minimize false positives, this dataset can be used to estimate differential expression at the gene-level for each brain tissue. More recently, Miller et al. have generated RNA-sequencing data from developing human brains. This dataset provides gene and exon-level reads per kilobase million (RPKM) mapped across the human genome and describes transcript isoforms associated with specific brain tissues.

These two datasets provide the most inclusive temporal and spatial map of brain transcription. The data shows that 84% of all genes are expressed in at least one brain structure [9]. Levels of gene expression in brain structures and developmental stages are strongly correlated between samples. The data also shows that there are significant differences in gene expression between different brain structures. Above all, the major finding of two studies is the transcriptome of the brain changes through development and age, with the largest change in gene expression occurring during the fetal stages. Clearly, by using these datasets, we have an opportunity to examine key molecular processes involved in early neurodevelopment [11,12] and assess the potential functional impact of DNA variations associated with neurodevelopmental disorders [13,14,15], including ASD [16,17,18].

Co-expression network analysis is a particularly powerful tool for examining high-throughput transcriptome data. Weighted gene correlation network analysis (WGCNA) is one method that is currently being used to investigate the correlation of genes across transcriptome samples [19]. The method is based on the principle that functionally similar genes are co-expressed across samples, where nodes are co-expressed genes and edges of the network are determined by the pairwise correlations between gene expressions [20]. From a set of samples or conditions, WGCNA can identify clusters of highly correlated genes, called co-expression modules, which are associated with a range of biological functions. Furthermore, this method provides the opportunity to find a hub (highly connected) gene in a module and relate modules to sample traits. 

In this study, we examine the spatio-temporal role of rare inherited variations in the developing human brain. We integrated two large post-mortem brain data sets to form a comprehensive transcriptome of the developing human brain and used co-expression analysis to characterize rare coding and regulatory variations identified from the genetic screening of ASD.

## 2. Materials and Methods 

### 2.1. Transcriptomic Datasets

Two transcriptomic datasets for developing human brains were obtained from Kang et al. (2011) and Miller et al. (2014). 

The dataset of Kang et al. was downloaded from the Gene Expression Omnibus (GEO) (ID: GSE25219), National Center for Biotechnology Information (NCBI), using biomaRt [21], an R software package. We excluded the brain samples with a low RNA integrity number (RIN) to avoid false-positives [22]. Subsequently, we selected a total of 1223 brain tissues with high quality, and then divided them into five groups according to their developmental stage:

(1) Stage 1—early to mid fetal (8–13 PCW; *n* = 206)
(2) Stage 2—mid to late fetal (14–38 PCW; *n* = 283)
(3) Stage 3—infancy and early childhood (birth–3 years; *n* = 130)
(4) Stage 4—mid-childhood to young adulthood (3–40 years; *n* = 279)
(5) Stage 5—young adulthood to late adulthood (40–60 years; *n* = 325)

The dataset of Miller et al. was downloaded from the Developmental Transcriptome of the BrainSpan [1]. After excluding low-quality brain samples, we selected samples from the early development stage (8 PCW to 45 months; *n* = 188). To examine the spatial association, we selected four brain regions, based on the transcriptional similarity of surrounding tissues [17]:

(1) Prefrontal and primary motor-somatosensory cortex-dorsolateral prefrontal cortex (DFC), primary motor cortex (M1C), medial prefrontal cortex (MFC), orbitofrontal cortex (OFC), primary somatosensory cortex (S1C), and ventrolateral prefrontal cortex (VFC);
(2) Primary visual cortex-superior temporal cortex cluster-primary auditory cortex (A1C), inferior temporal cortex (ITC), inferior parietal cortex (IPC), superior temporal cortex (STC), and primary visual cortex (V1C);
(3) Striatum-hippocampus-amygdaloid cluster-amygdala (AMY), hippocampus (HIP), and striatum (STR);
(4) Mediodorsal nucleus of thalamus-cerebellar cortex cluster-locomotion cerebellar cortex (CBC) and mediodorsal nucleus of the thalamus (MD).

For both datasets, we used the gene expression profile of protein-coding genes annotated in ENSEML65 (*n* = 15,585). 

### 2.2. WGCNA Analysis

The co-expression network analysis was performed using the R WGCNA package [19], as previously described [23]. To calculate the power that satisfied scale-free criteria, we measured pairwise correlation-based adjacency values of gene expressions in the given datasets. Then, we estimated neighborhood similarity among genes and identified co-expression gene modules using hierarchical clustering. The minimum module size was set to 20 genes, and the height for merging modules was set to 0.15, which required at least 15% dissimilarity among modules. Co-expressed gene modules were summarized by their module eigengene (ME) values, defined as the first principal component of the standardized expression values.

### 2.3. Characterization of Co-Expressed Gene Modules

We characterized the co-expressed gene modules using the Gene Ontology (GO) analysis at ClueGO, a CytoScape plug-in [24]. We used GO terms for biological process, based on experimental evidence. The statistical significance of GO terms was calculated by the hypergeometric test and then adjusted using a Bonferroni step-down correction for false discovery rate <0.05.

### 2.4. Enrichment Test of Rare Variations in Co-expressed Gene Modules

To compare enrichment of rare variations in co-expressed gene modules, we define rare variations as (1) Rare coding variants (SNPs and indels), (2) Rare regulatory variants (SNPs and indels), (3) Rare DNVs (including *de novo* SNPs and indels), and (4) Rare copy-number variations (CNVs). We obtained genes with rare coding and regulatory variants (SNPs and indels) identified from our previous WES studies [25,26]. Considering our aim is to examine rare inherited variations, we compared the enrichment of genes with rare de novo variants (DNVs) identified by a recent large family-based WES study [27]. Rare loss-of-function DNVs for control backgrounds were selected from siblings of ASD cases. For the gene set of rare CNVs, we used genes of rare CNVs from the microarray analysis of SSC families [28]. The control gene set comprised randomly selected genes of CNVs found in a general population, obtained from the Database of Genomic Variants (DGV) [29]. 

Due to a difference in the size between the case and control gene sets, we randomly sampled 800 genes (approximately 80% of genes found in cases) with 10,000 iterations to calculate resampling statistics for enrichment tests using a binomial distribution with standardized z-score. We confirmed that the rate of false-positives, as determined by z-scores, was higher in controls than from random sampling. 

## 3. Results

### 3.1. Co-Expression Networks of Five Developmental Stages of The Human Brain

To determine if there were transcriptional differences during early brain development, we created a co-expression network of five developmental stages of the human brain. Here, we performed a signed co-expression network analysis using WGCNA R software. WGCNA provides a landscape of gene expression patterns across given samples and characterizes the features of co-expression gene modules. It basically measures topological overlaps derived from pairwise correlation-based adjacency values to investigate the neighborhood similarity among genes. Then, it identifies co-expression gene modules by the hierarchical clustering method. 

**Figure 1:**
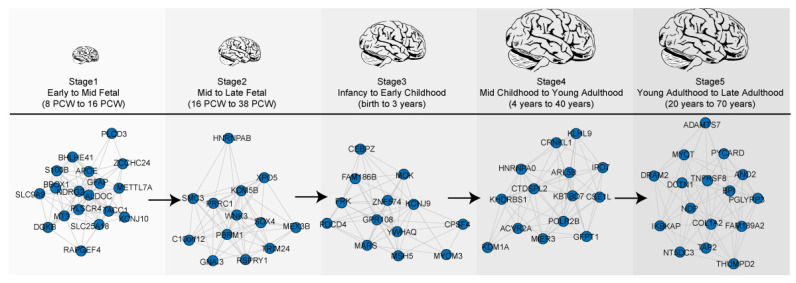
Temporal dynamics of gene expression during brain development. Five co-expression networks are highlighted by changes in co-expressed genes associated with chromatin/transcriptional regulation over developmental stages.

For this, we chose the dataset of Kang et al. (2011) as they have the largest number of samples from the fetal to late adulthood stage brains (Table 1). Five co-expression networks were constructed for each developmental stage comprised of 7–15 gene modules. Most of these were characterized on the basis of biological function (functional modules), i.e., nucleic acid metabolic process, cell communications, neurogenesis, synaptic development, protein synthesis, or immune system, and were shown to have hundreds of module genes (Table S1). Irrespective of occurring in the same functional module, we found different genes were involved in each developmental stage (Figure 1). This suggests that biological function is dynamically modulated via transcriptional changes over these neurodevelopmental stages.

### 3.2. Enrichment of Rare Variations in Functional Modules of Five Neurodevelopmental Stages 

**Figure 2:**
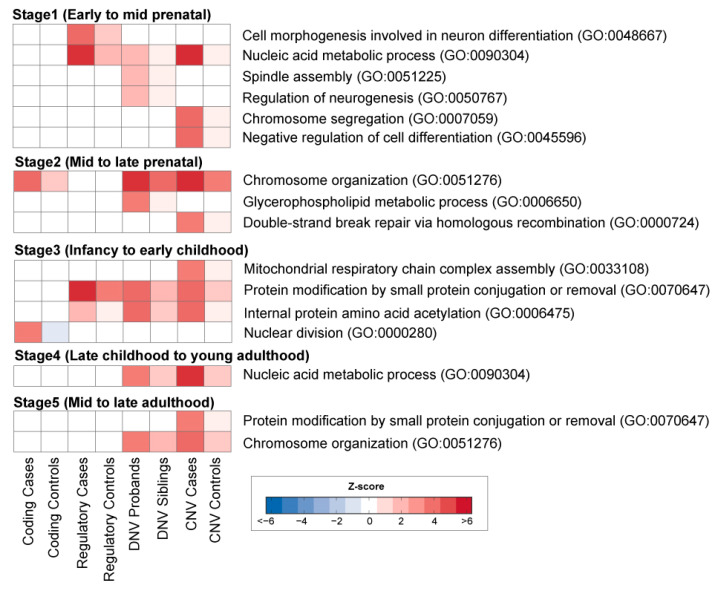
WGCNA analysis of human brain development. Heatmaps (bottom) show enrichment of rare variations in the co-expression gene modules. Heatmap (blue to red) reflects a significant association of variants as determined by z-score enrichment.

Rare variations associated with ASD were mostly enriched in co-expressed gene modules of the Stages 1–3 (42%; 13 out of 31 modules) compared to those from Stages 4–5 (19%; 3 out of 16 modules) (Figure 2). This suggests rare DNA variations associated with ASD likely occur in genes that are predominately expressed during early brain development. This data is consistent with the idea that ASD is a neurodevelopmental disorder with a prenatal trajectory.

Interestingly, some modules were enriched with multiple types of rare variants, whereas others were specifically enriched with only one type of rare variants. For instance, throughout early fetal to early childhood, all four types of rare variations in ASD cases were shown to be involved in transcriptional processes, including nucleic acid metabolic processes or chromosome organization [30]. In contrast, some modules in Stage 1 (early to mid fetal) were exclusively associated with rare regulatory variants involved in cell morphogenesis and neuron differentiation (GO:0048667). In contrast, rare coding variants were enriched in the module of nuclear division (GO:0000280) in Stage 3 (infancy to early childhood). 

Compared to rare coding and regulatory SNP variants, DNVs and CNVs were found to affect multiple pathways in the early (fetal to early childhood) neurodevelopmental stages. We found DNVs were enriched in the module for the regulation of neurogenesis (GO:0050767), spindle assembly (GO:0051225), and glycerophospholipid metabolic process (GO:0006650), whereas CNVs were enriched in the module of chromosome segregation (GO:0007059), negative regulation of cell differentiation (GO:0045596), double-strand break repair via homologous recombination (GO:0000724), and mitochondrial respiratory chain complex assembly (GO:0033108). Taken together, our analyses suggest early brain development is likely affected by a number of rare variations associated with ASD. 

### 3.3. Co-Expression Networks of the Early Neurodevelopment

**Figure 3:**
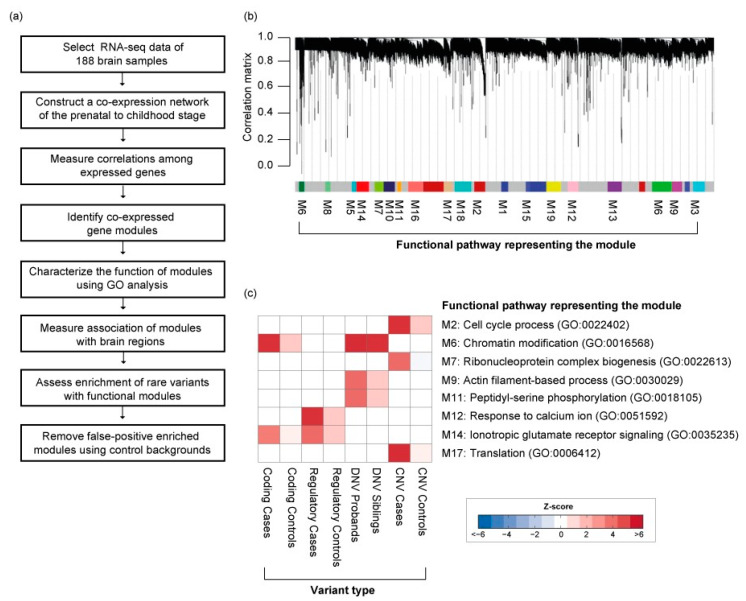
Co-expressed gene network in the early neurodevelopment stage. (**a**) Detailed workflow for the construction of the co-expression gene network. (**b**) Co-expression gene modules enrichment with rare variations. (**c**) Type of rare variation found enriched in co-expressed gene modules.

To elucidate the spatial and temporal significance of rare DNA variations occurring in common biological functions during the early development of the brain, we used the BrainSpan developmental transcriptome [1], generated by deep RNA-sequencing assay for 188 high-quality brain samples (Figure 3a). We applied WGCNA on RNA-sequencing data from these samples, as previously described. In this analysis, a “soft threshold” power of 24 was used to meet the scale-free criteria of the co-expression network. We chose co-expression gene modules with the following conditions: (1) at least 10 genes in the co-expression gene module; and (2) at least a 75% correlation value among genes of the module. As a result, we found a total of 17 co-expressed gene modules associated with biological functions during early neurodevelopment stages (Figure 3b) (Table S2).

### 3.4. The Functional Role of Rare Variations in the Early Neurodevelopmental Stage

**Figure 4:**
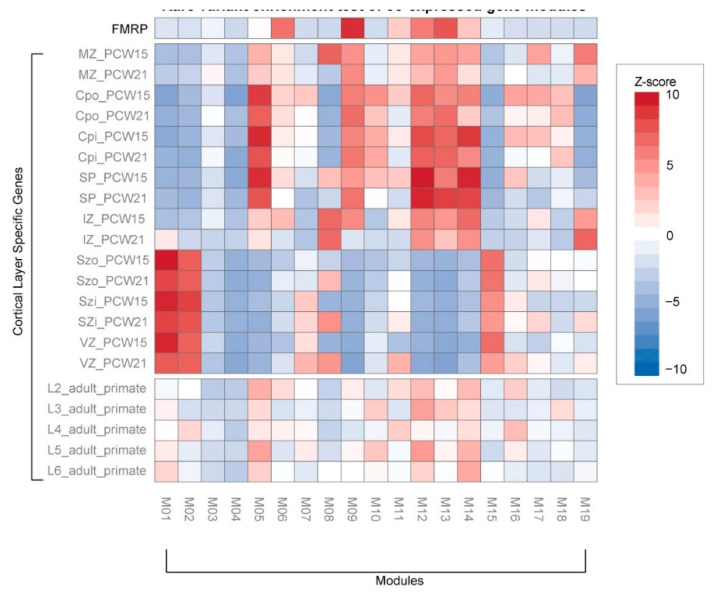
Rare variant enrichment test of co-expressed gene modules. We compared module genes with the FMRP target genes and other genes previously associated with the cortical layer. Cortical layer genes were selected from different laminae: marginal zone (MZ), outer/inner cortical plate (CPo/CPi), subplate (SP), intermediate zone (IZ), outer/inner subventricular zone (SZo/SZi), ventricular zone (VZ), and adult cortical layers 2–6 (L2–6). The heatmap of standardized z-scores (one-tailed binomial test) indicates significance (blue to red) of co-expressed genes modules associated with developmental stages of the brain.

Rare variations associated with ASD were significantly enriched in 8 co-expressed gene modules (Figure 3c). Rare coding variants and DNVs were enriched in module 6 (M6), namely, chromatin modification (GO:0016568) and chromatin organization (GO:0006325). This module significantly overlaps with the network of previously reported rare DNVs associated with the early-mid fetal stage brain development [18]. In contrast, we found rare regulatory variants were significantly enriched in M12, the response to calcium ion (GO:0051592) and ion transport (GO:0006811), including some key genes encoding voltage-gated calcium channels (*CACNA2D1*, *CACNB4*). Interestingly, both M6 and M12 significantly overlap with Fragile X mental retardation proteins (FMRP; *p*-value < 1.0^−10^; Figure 4) [31]. Rare CNVs are enriched for M7 and M17, containing genes in translation and ribosomal complex. Genes affected by DNVs are mostly found in M9 and M11, which are involved in actin filament-based process and peptidyl-serine phosphorylation. Taken together, the evidence suggests these rare inherited variants may be involved in synapse development and maturation during the early development of the brain.

In contrast, rare CNVs appear to have very different functional roles (Figure 3c). We found CNVs were significantly enriched in M2, namely, cell cycle process (GO:0022402), ribonucleoprotein complex biogenesis (GO:0022613), and translation (GO:0006412). This module is significantly associated with deep layers in the fetal cortex and juxtaposed to regions associated with M12 and M14 (Figure 4). This suggests the role of CNVs in early neurodevelopment may be different from rare inherited or DNVs.

Considering the above, patterns of gene expression overlaid with rare coding and regulatory variations suggest that frontal and somato-motor neocortical development during the first trimester is a critical period of brain development that may initiate a pathophysiological trajectory for ASD. Furthermore, based on gene ontology classification, many of the co-expressed genes of rare variations are involved in synaptic plasticity, development, and neuronal differentiation. Therefore, our data are consistent with previous studies that suggest disruptions in neural circuit formation and neuronal plasticity mechanisms as important functions underpinning ASD [17,18] and a focus for further investigation.

### 3.5. Neurogenesis Affected by Rare Inherited Coding and Regulatory Variants

**Figure 5:**
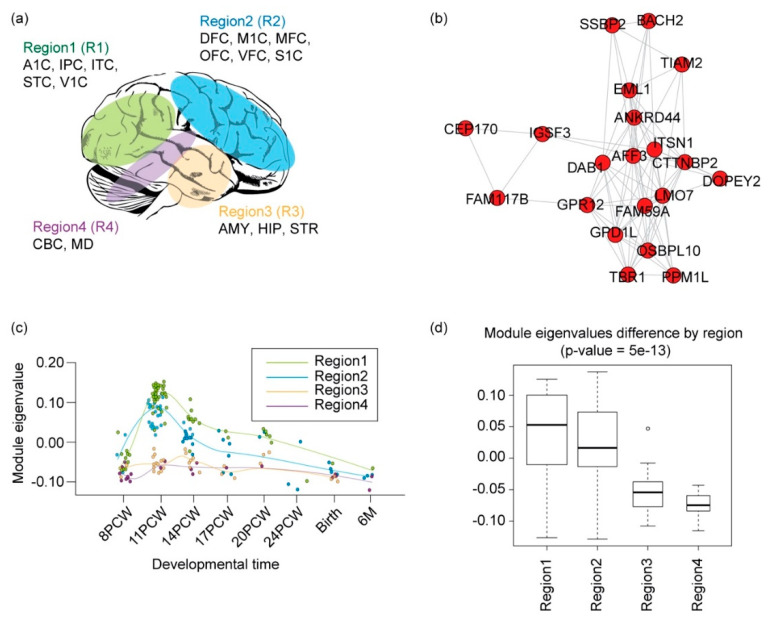
Functional convergence of rare coding and regulatory variants in the mid-fetal neocortex. (**a**) An anatomical selection for WGCNA. Shown are four brain regions grouped according to similarity of gene expression pattern (Willsey et al. 2013) [3]. (**b**) Network of co-expressed gene module 14, including genes involved in ionotropic glutamate receptor signaling (GO:0035235) and generation of neurons (GO:0048699). (**c**) Eigenvalues of gene modules through development. The circles represent the samples from each region. Regions include four regional groups from Figure 5a, and use the same color code for a regional group. The *x*-axis indicates the developmental time, and the *y*-axis indicates the module eigenvalue of the samples for each developmental time. PCW: post-conception weeks; M: months. (**d**) Eigenvalues of the gene modules across the four brain regions.

Rare coding and regulatory variants were enriched in module 14 (M14), mostly associated with ionotropic glutamate receptor signaling pathway (GO:0035235) and generation of neurons (GO:0048699) (Figure 5b). For instance, five ASD cases from three families were affected by missense variants of *CTTNBP2*, an important ASD candidate gene [27,32] that regulates dendritic spinogenesis via the glutamatergic pathway [33]. We also found that three ASD cases had rare indels in the promoter region of *Tbr1*, frequently reported in ASD [34] and known to contribute to abnormal cognition [35]. This gene functions by modulating the production of neurons and oligodendrocytes [36] and is more generally involved in excitatory synaptic transmission and glutamatergic pathways [35]. This module (M14) is particularly interesting because it is significantly associated with neocortex regions during early fetal development (11–14 PCW; Figure 5c) and is enriched in superficial cortical layers at early-mid fetal periods (15–21 PCW; Figure 4). To further validate the top pathway genes from the module, we retrieved their protein expression from the Human Protein Atlas [37,38]. As a result, we were able to validate the presence of top pathway genes and find a strong correlation between gene and protein expression (R^2^ = 0.74; *p*-value = 2.0^−4^) that could be a potential biomarker for further research. Taken together, rare coding and regulatory variants disrupt cortical–cortical connectivity during early neurodevelopment in ASD.

## 4. Discussion

Recent advances in transcriptome analyses using human post-mortem tissue have highlighted intricate molecular patterns involved in human brain development. Moreover, brain transcriptome data has revealed a remarkable flux of gene expression changes during prenatal and early postnatal brain development [7,8]. These data are now providing critical insight into the biological trajectories of specific genes involved in key neurodevelopmental processes [39,40].

One emerging theme is the idea that early neurodevelopment can be perturbed or disrupted by any one of hundreds of DNVs [18]. Two previous studies performed co-expression networks analysis and demonstrated that genes with ASD-associated DNVs are highly expressed during early neurodevelopment (mid fetal to early infancy) [17,18]. These studies rigorously identified genes with DNVs occurring in the same dataset using the same analysis pipeline. The observation by Willsey et al. (2013) that genes with DNVs are expressed during the mid-fetal (PCW 10–24) and have differentially expressed enrichment in the superficial layers of cortical projection neurons in the neocortical region of the brain is of particular significance to ASD, an observation supported by our study.

Aside from DNVs, it was important to ascertain whether rare inherited variations affect early neurodevelopment. Kang et al. (2011) provides evidence that both rare inherited and *de novo* variations associated with ASD are frequently enriched in functional pathways involved in early neurodevelopment. The data again underscores how rare variations with low allele frequencies persist as a consequence of purifying selection—these variations persist in the population as a consequence of recessive inheritance, incomplete penetrance, or spontaneous variations [41].

Our analyses, for the first time, demonstrate the functional relationship of rare inherited variations associated with ASD in the developing brain. Indeed, inherited variations are known to account for 40% of the risk for ASD [42], with each allele contributing arguably a small fraction to the total risk [43]. We also observed that different types of rare variations have different roles in the early neurodevelopment. When examining a more comprehensive dataset, we see functional differences between rare regulatory and coding variants. Our data show that chromatin regulation was affected by both rare coding variants and DNVs, supporting previous evidence that DNVs are involved in chromatin regulation and early neurodevelopment in ASD [18]. In contrast to rare coding variations, we find rare regulatory variations involved in early development occur in synaptic genes (calcium channel or glutamate signaling). Many synaptic genes (loss-of-function coding variations) have been previously associated with ASD, therefore, rare non-coding regulatory variations may contribute to genetic risk [44,45].

## Figures and Tables

**Table 1:** Summary of weighted gene correlation network analysis (WGCNA).

	Stage1 *	Stage2 *	Stage3 *	Stage4 *	Stage5 *	Early Neurodevelopment **
Number of brain samples	257	292	130	279	325	188
Number of co-expressed gene modules	15	7	11	9	11	19
Number of functional modules	13	7	11	7	9	17
Number of genes per module	391	1992	680	1018	795	472

* The dataset was obtained from Kang et al. [2]. ** The dataset was obtained from Miller et al. [1]. Detailed functional modules are described in Table S1.

## Supplementary Materials

The following are available online at https://www.mdpi.com/2073-4425/11/5/535/s1, Table S1: The list of co-expressed gene modules in the WGCNA. Table S2: Co-expression modules of the early development. Table S3: Protein and gene expression of ionotropic glutamate receptor signaling (GO:0035235) and generation of neurons (GO:0048699) pathways.
