# Supplementary material for: Spatio-Temporal Roles of ASD-Associated Variants in Human Brain Development
Source: Genes (Basel). 2020 May 11;11(5):535. doi: 10.3390/genes11050535 (PMC7291218; doi:10.3390/genes11050535)
Supplement: Supplementary file 1 [file genes-11-00535-s001.pdf]

Supplementary Table 1. The list of co-expressed gene modules in the WGCNA

Stage	Module number	Pathway Name	Pathway ID	Adj. p-value	No. Module genes
**Stage1**	1	Nucleic acid metabolic process	GO:0090304	3.13E-17	593
**Stage1**	2	Regulation of immune system process	GO:0002682	9.87E-07	2116
**Stage1**	3	Cell morphogenesis involved in neuron differentiation	GO:0048667	3.38E-03	708
**Stage1**	4	Regulation of neurogenesis	GO:0050767	2.73E-03	109
**Stage1**	5	Cell communication	GO:0007154	8.10E-04	439
**Stage1**	6	Positive regulation of cell growth	GO:0030307	4.54E-04	104
**Stage1**	7	Spindle assembly	GO:0051225	3.80E-03	120
**Stage1**	8	Cytokinesis	GO:0000910	2.20E-03	92
**Stage1**	9	Negative regulation of cell differentiation	GO:0045596	1.70E-04	81
**Stage1**	10	Cellular potassium ion homeostasis	GO:0030007	2.74E-04	140
**Stage1**	11	Protein O-linked glycosylation	GO:0006493	3.84E-04	93
**Stage1**	12	Eye development	GO:0001654	3.51E-04	122
**Stage1**	13	Chromosome segregation	GO:0007059	1.09E-19	371
**Stage1**	14	NA	NA	NA	239
**Stage1**	15	NA	NA	NA	536
**Stage2**	1	Stem cell differentiation	GO:0048863	1.57E-03	148
**Stage2**	2	Chromosome organization	GO:0051276	9.81E-34	4376
**Stage2**	3	Defense response	GO:0006952	2.19E-11	3804
**Stage2**	4	Double-strand break repair via homologous recombination	GO:0000724	2.66E-04	403
**Stage2**	5	Glycerophospholipid metabolic process	GO:0006650	5.24E-03	183
**Stage2**	6	Signal transduction	GO:0007165	6.05E-09	4512
**Stage2**	7	Mitochondrial respiratory chain complex assembly	GO:0033108	6.62E-04	516
**Stage3**	1	Mitochondrial respiratory chain complex assembly	GO:0033108	2.52E-03	734
**Stage3**	2	Nuclear division	GO:0000280	7.03E-03	990
**Stage3**	3	Cytoplasmic sequestering of transcription factor	GO:0042994	5.37E-04	199
**Stage3**	4	Internal protein amino acid acetylation	GO:0006475	8.75E-03	918
**Stage3**	5	Negative regulation of gene expression	GO:0010629	2.52E-03	287

**Stage3**	6	DNA metabolic process	GO:0006259	7.13E-03	618
**Stage3**	7	Establishment of protein localization to endoplasmic reticulum	GO:0072599	3.82E-04	250
**Stage3**	8	Protein modification by small protein conjugation or removal	GO:0070647	1.62E-06	749
**Stage3**	9	Regulation of epithelial cell proliferation	GO:0050678	9.92E-04	215
**Stage3**	10	Leukocyte cell-cell adhesion	GO:0007159	2.69E-05	1535
**Stage3**	11	Intracellular cholesterol transport	GO:0032367	4.83E-03	981
**Stage3**	12	NA	NA	NA	233
**Stage3**	13	NA	NA	NA	1201
**Stage3**	14	NA	NA	NA	205
**Stage4**	1	Nucleic acid metabolic process	GO:0090304	2.22E-18	1173
**Stage4**	2	Positive regulation of immune system process	GO:0002684	1.42E-13	3193
**Stage4**	3	Regulation of membrane potential	GO:0042391	6.89E-03	1000
**Stage4**	4	Monovalent inorganic cation transport	GO:0015672	1.22E-03	196
**Stage4**	5	Anatomical structure morphogenesis	GO:0009653	9.04E-03	348
**Stage4**	6	Cellular macromolecule catabolic process	GO:0044265	1.29E-03	674
**Stage4**	7	Nervous system development	GO:0007399	1.23E-03	541
**Stage5**	1	Chromosome organization	GO:0051276	2.22E-14	900
**Stage5**	2	Regulation of immune system process	GO:0002682	1.17E-11	3646
**Stage5**	3	Monovalent inorganic cation transport	GO:0015672	1.49E-04	865
**Stage5**	4	Positive regulation of epithelial cell migration	GO:0010634	1.13E-03	110
**Stage5**	5	Cell development	GO:0048468	2.64E-04	177
**Stage5**	6	Regulation of endothelial cell proliferation	GO:0001936	1.84E-03	187
**Stage5**	7	Regulation of protein localization to plasma membrane	GO:1903076	8.95E-04	425
**Stage5**	8	Cell cycle	GO:0007049	1.18E-19	67
**Stage5**	9	Protein modification by small protein conjugation or removal	GO:0070647	4.76E-07	779
**Stage5**	10	NA	NA	NA	446

*The dataset from Kang *et al*. (2011) was used in this analysis

Supplementary Table 2. Co-expression modules of the early development

Module	Representative GO term	GO ID	Adj. p-value	No. Genes in the module
**Module1**	Sulfur compound metabolic process	GO:0006790	3.93E-003	859
**Module2**	Cell cycle process	GO:0022402	1.31051E-31	1393
**Module3**	NA	NA	NA	442
**Module4**	Cellular response to cadmium ion	GO:0071276	1.85E-04	175
**Module5**	Positive regulation of mitochondrion organization	GO:0010822	1.24E-04	165
**Module6**	Chromatin modification	GO:0016568	1.54E-20	717
**Module7**	Ribonucleoprotein complex biogenesis	GO:0022613	4.00E-07	338
**Module8**	Cell activation	GO:0001775	4.49E-13	202
**Module9**	Actin filament-based process	GO:0030029	3.12E-03	379
**Module10**	Proteasomal protein catabolic process	GO:0010498	1.02E-03	414
**Module11**	Peptidyl-serine phosphorylation	GO:0018105	6.76E-04	150
**Module12**	Response to calcium ion	GO:0051592	4.61E-04	385
**Module13**	NA	NA	NA	514
**Module14**	Ionotropic glutamate receptor signaling pathway	GO:0035235	1.05E-03	466
**Module15**	Determination of liver left/right asymmetry	GO:0071910	9.65E-03	346
**Module16**	Cellular macromolecule metabolic process	GO:0044260	8.54E-06	528
**Module17**	Translation	GO:0006412	4.23E-15	326
**Module18**	Mitochondrion organization	GO:0007005	1.21E-05	619
**Module19**	Cardiovascular system development	GO:0072358	2.46E-13	543

Supplementary Table 3. Protein and gene expression of ionotropic glutamate receptor signalling (GO:0035235) and generation of neurons (GO:0048699) pathways

Gene/Protein	Location	Gene Expression	Protein Expression
AFF3	Cerebral cortex	5.8	18.6
ANKRD44	Cerebral cortex	1.1	10.9
BACH2	Cerebral cortex	0.9	4.2
CEP170	Cerebral cortex	8.5	54.6
CTTNBP2	Cerebral cortex	7.2	17.1
DAB1	Cerebral cortex	0.2	7.2
DOPEY2	Cerebral cortex	3.9	11.1
EML1	Cerebral cortex	7	20.6
FAM117B	Cerebral cortex	3.5	9.7
FAM59A (GAREM1)	Cerebral cortex	3.7	16.6
GPD1L	Cerebral cortex	15.4	48
GPR12	Cerebral cortex	5	9.6
IGSF3	Cerebral cortex	3.1	7.8
ITSN1	Cerebral cortex	16.9	25.4
LMO7	Cerebral cortex	15.9	48.9
OSBPL10	Cerebral cortex	2.7	5.9
PPM1L	Cerebral cortex	8.6	33.7
SSBP2	Cerebral cortex	21.2	40.9
TBR1	Cerebral cortex	25.4	36.8
TIAM2	Cerebral cortex	13.8	21.8
